# Teaching as a system: COVID-19 as a lens into teacher change

**DOI:** 10.1007/s10649-021-10107-3

**Published:** 2021-10-25

**Authors:** Domenico Brunetto, Giulia Bernardi, Chiara Andrà, Peter Liljedahl

**Affiliations:** 1 Politecnico Di Milano, Milan, Italy; 2 Università del Piemonte Orientale, Vercelli, Italy; 3 Simon Fraser University, Burnaby, Canada

**Keywords:** Mathematics-related affect, University math professors, Online teaching, Teacher change, Case study research

## Abstract

In the spring of 2020, schools and universities around the world were closed because of the COVID-19 pandemic. The relative lockdown affected more than 1.5 billion learners as teachers and students sheltered at home for several weeks. As schooling moved online, teachers were forced to change how they taught. In the research presented here, we focus on university mathematics professors, and we analyze how their practice, knowledge, and beliefs intertwine and change under these circumstances. More specifically, the context of the pandemic and the relative lockdown provides us with the experimental basis to argue that the new practice affected both knowledge and beliefs of mathematics teachers and that practice, knowledge, and beliefs form a system. Being part of a system, the reactions to change in practice can be of two types, namely, the system as a whole tries to resist change, or the system as a whole changes — and it changes significantly. The research presented here proposes a model for describing and analyzing what we called a teaching system and examines three cases that help to better depict the systemic nature of teaching.

## Introduction

During the pandemic spread at the beginning of 2020, almost all schools and universities were closed, and teachers had to rapidly re-organize their practice to provide online learning environments for their students (Arum & Stevens, 2020; Gülbahar & Adnan, 2020). Teachers transitioned, created, and implemented online teaching even if they did not feel adequately prepared to do so or previously had little interest for online teaching (Hechinger & Lorin, 2020; McMurtrie, 2020). In this paper, we address the specificity of mathematics teaching as a twofold issue. First, mathematics is traditionally deemed to be strongly connected to content, and, as a consequence, teaching mathematics is traditionally connected to the transmission of knowledge, namely, facts to be remembered and skills to be acquired. From this perspective, the transition from in-class to online teaching could be straightforward for a mathematics teacher. However, mathematics education research has provided evidence that learning mathematics is more effective, more lasting, and deeper when students participate in the process of learning, being involved in problem-solving activities and interaction with peers (Stein et al., 2008). Regardless of the approach, however, teaching methods are heavily dependent on both teachers’ beliefs and the context in which that teaching takes place (Skott, 2001). Second, unlike other disciplines such as biology or chemistry or physics, which require physical environments and tools for the students’ interaction, mathematics has the unique feature of being primarily concerned with abstract objects. So, in theory, the interaction with its “tools” can take place even in distance/online learning. Taken together, online teaching can either provoke or discourage more participative mathematics lessons. Within this study, we investigate whether the transition to online teaching, which represented a change in mathematics teachers’ practices, also provoked a change in their beliefs about their mathematics teaching methods.

We have known for a long time that there is a connection between teachers’ practice, their knowledge (Ben-Peretz, 2011), and their beliefs (Fosnot, 1989; Skott, 2001). For the most part, prior research has considered teaching practice as a consequence of teachers’ knowledge and beliefs, and, therefore, changes in teachers’ practice were achieved through changes to their knowledge (Ball, 1988; Feiman-Nemser & Featherstone, 1992) and beliefs (Leder et al., 2002; Rolka et al., 2006). One voice that stood in opposition to this idea was Guskey (1986), who showed that teacher beliefs could change as a result of changes to their practice, and the mechanism of that change was evidence of students’ improved learning. Liljedahl (2016) extended this idea through his notion of a first-person vicarious experience by showing that changes in teachers’ practice can lead to changes in beliefs, not only through evidence of student learning, but also through evidence of student enjoyment and behavior in the learning setting. In this work, we push these ideas further and document the changes in teacher’s beliefs and knowledge within the recent COVID-19 upheaval, where circumstances necessitated changes in practice. To that end, we collected data regarding the experience of mathematics university professors, who had to move their practice online in the spring of 2020.

### Theoretical framework: teacher practice, knowledge, and beliefs

Teacher practice is the work that teachers do when they carry out their professional tasks (Da Ponte & Chapman, 2006). According to Dougherty (1990), it includes, but is not limited to, lesson development, selection of examples, and choice of lesson format. According to Khisty et al. (1990), it also includes the language used, the nature of the classroom discourse, and the tasks proposed. We can also add to the list the use of digital tools, the lesson planning, and the choice of the classroom setting.

Shulman (1986, 1987) developed the idea that teachers’ knowledge goes beyond their knowledge of the content (that is, content knowledge), co-joining with how they teach it to form the specialized knowledge that he called pedagogical content knowledge (PCK). According to Shulman (1986), PCK is a form of knowledge building upon, but not the same as, subject matter knowledge and knowledge of general principles of pedagogy, contextualized in a particular classroom setting (Hurrell, 2013). Researchers have expanded on Shulman’s ideas to include task knowledge (Chapman, 2013; Johnson et al., 1988), technology knowledge (Mishra & Koehler, 2006; Niess, 2005), the knowledge quartet (Rowland et al., 2009), and so on, culminating in the decade-long comprehensive research program by Ball et al. (2005) embodied in the *knowledge for teaching* model. In the following, we mean knowledge as “a body of professional knowledge that encompasses both knowledge of general pedagogical principles and skills and knowledge of the subject matter to be taught” (Ben-Peretz, 2011, p.8), including also technological knowledge (and in particular knowledge of digital resources for teaching online).

Parallel to teacher knowledge, a central role for beliefs emerges: that is, not only what a teacher knows, but also what a teacher believes, impacts what and how they teach (Ball, 1988; Lortie, 1975). Green (1971) investigates the formation of beliefs, in general, and the formation of what he called belief clusters — a metaphor for talking about the fact that “beliefs come always in sets or groups, never in complete independence of one another” (p. 41). These systems are organized according to the quasi-logical relations between the beliefs, the psychological strengths with which each belief is held, and the ways in which beliefs cluster. Although he does not explicitly link these ideas to the broader context of systems theory (Buckley, 1967; Von Bertalanffy, 1968, 1975), it is difficult to believe, given the time period, that he was not influenced by this research. As such, Green’s idea of a belief cluster is actually a belief system. And like all systems, in a belief system, all the features reinforce each other. If one feature is changed, the system will rush to “repair the damage” (Stigler & Hiebert, 1999). This drive to repair themselves has formed the idea that beliefs are stable (*ibidem*).

By nature, beliefs are also “hidden” (Leder et al., 2002), and they can be studied “only by inferring them from how people think and act” (Lester, 2002, p.346). To this respect, affect-related research has provided evidence in the last decades that beliefs have observable behavioral consequences (Di Martino & Zan, 2011), and a change in a teacher’s beliefs is likely to result in a change in their practice (Leder et al., 2002). This leads us to conjecture that practice, belief, and knowledge represent the main components to understand and model both teaching and changes in teaching, and that they form a system: the teaching system.

### Teaching system

Like beliefs, the last several decades of research on teaching have built up a perception that teaching practice is stable and difficult to change (Beswick, 2006). However, there are several examples from literature (Guskey, 1986; Kleickmann et al., 2013; Lee et al., 2007), and in the lived experiences of many researchers in mathematics education who teach in professional development courses, that change is not only possible, but that it can happen quite quickly (Liljedahl, 2010). Guskey (1986) showed that changes in practice occasion change in beliefs, reporting very clearly that when changes in teacher practice occur, and resultant changes to student learning are observed, the associated beliefs about teaching and learning then change. This suggests that practice, beliefs, and knowledge are inter-connected in a systemic way, as change in one component provokes a change in the others.

We loosely use the term “change,” which has different meanings and takes up different forms if it applies to practice, or knowledge, or beliefs. For instance, if we focus on belief change, “change” might mean that a new belief is formed, for example, as a result of a new experience, and replaces another one (for a review on how beliefs can be formed from experiences, see, e.g., Furinghetti & Pehkonen, 2002). Alternatively, new connections in the belief system may emerge or cluster in a new way, or an implicit belief may become explicit, and the belief system adjusts to the new situation. On the other hand, events like a professional development course, the study of a new topic, or sharing an experience with a colleague may provoke a change in knowledge wherein new facts are acquired and become part of a teacher’s repertoire. Finally, a change in practice might mean to change the way a lesson is planned (for example, planned in finer details or planned with the aid of a new tool) and/or carried out (for example, introducing an interactive whiteboard). There could be a modification of students’ roles or the use of different tools and so on.

A prominent method for evoking change in teachers is by involving them as learners of mathematics (and mathematics pedagogy), usually immersed in a constructivist environment (Ball, 1988; Feiman-Nemser & Featherstone, 1992). This means that a change in teachers’ practice can provoke a change in their beliefs. For example, in the recent context of the COVID-19 pandemic, Cutri et al. (2020) showed that some teachers were more comfortable online than in person, and this can imply that a new practice had an impact on these specific teachers’ beliefs. On the other hand, as discussed above, teachers’ practice is strongly influenced by their beliefs (Ball, 1988; Leder et al., 2002; Lortie, 1975). Taken together, we can conclude that practice and beliefs are mutually influenced by each other.

**Fig. 1:**
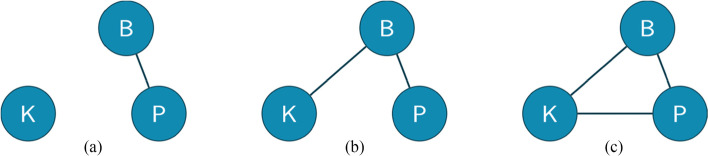
The development of the teaching system

We propose to model this reciprocal influence as a connection between practice (labeled as “P”) and the belief system (labeled as “B”). Furthermore, we consider P and B as nodes in a graph, and we draw an undirected edge (i.e., without arrows) between them to represent their connection and reciprocal influence (see Fig. 1a).

Another method for producing changes in belief structures has emerged from the work of Rolka et al. (2006), in which it has been shown that preservice teachers’ experiences with mathematical discovery have a profound and immediate transformative effect on their beliefs. When teachers’ knowledge (that we consider as a node labeled “K”) about mathematics is changed, a change in their beliefs might be provoked; thus, these two elements are mutually connected. We can add an edge between K and B in the graph we are building (see Fig. 1b).

Finally, there is a connection between practice and knowledge, as recalled in the previous paragraphs (Hurrell, 2013; Shulman, 1986, 1987). Thus, we can add an edge between the elements P and K in Fig. 1c. The result of such a process is a connected graph (Newman, 2010), that is, a graph where each node is connected to at least another node, as it has been employed in a mathematics education context by Liljedahl (2018).

As a result, we model all these connections in a single connected graph that we call a *teaching system* (see Fig. 1c). We use the term *system* to refer to “a set of elements standing in interaction” (Von Bertalanffy, 1968, p.39).

### Research problematique

The peculiarity of the COVID-19 pandemic is that teacher practice has been forced to change: teachers had to adjust their teaching in order to provide digital (in place of in-classroom) teaching to their students. In Cutri et al.’s (2020) words:

During the early months of the year 2020, faculty around the world had to transition their courses online under circumstances that typical online course development does not have to face. Those circumstances were (1) a need to rapidly, with little to no preparation, transition instruction online; (2) execute the transition online and subsequent online instruction under traumatic conditions of a pandemic; and (3) pursue extended online teaching with little to no information regarding if this transition to online teaching will be temporary or more permanent. We assert that these three factors constitute crisis online course transitioning and teaching as opposed to conventional online course transitioning and teaching (p.3)

For us, this represents an opportunity to explore how a forced change in practice has an effect (or not) on beliefs and knowledge and for exploring the P–B–K system as a whole.

If our conjecture on the systemic nature of teaching holds true, then, when a change in one of its three elements occurs, either the system as a whole tries to resist the change and to repair the damage, maintaining its status as it was before the perturbation (for example, a new knowledge, which is in contrast with the old ones, is acquired but is ignored so as the system does not change), or the system as a whole changes, and perturbation propagates through the other elements (for example, new knowledge is acquired and the teacher implements it in practice, with adjustments also in beliefs). Specifically, we investigate if a change in practice can direct us to consider teaching as having a systemic nature and, thus, if we can observe either scenario mentioned above.

## Methodology

### The COVID-19 lockdown as change in practice

**Fig. 2:**
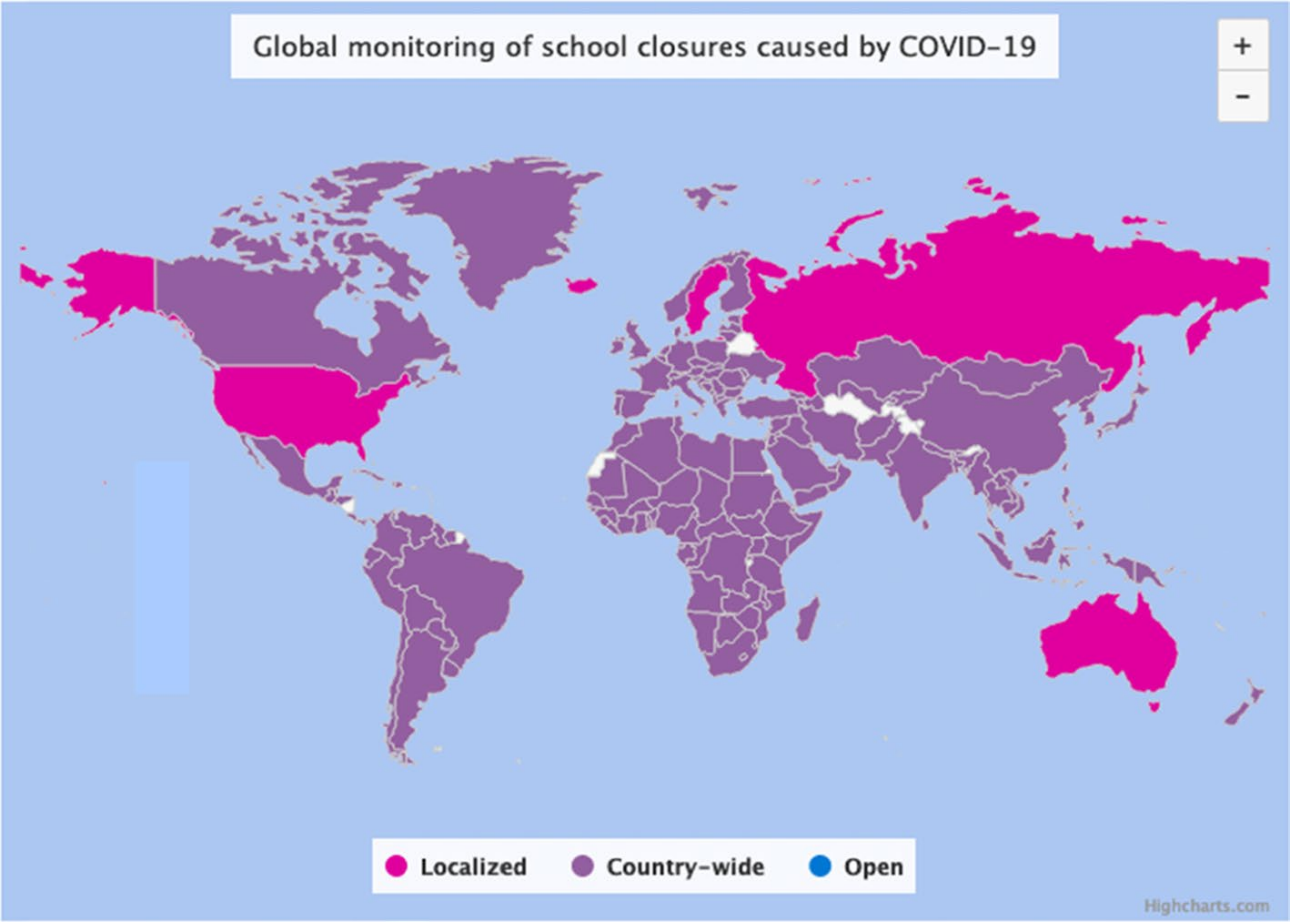
The school closures in April 2020. Source: https://en.unesco.org/covid19/educationresponse

The COVID-19 lockdown affected up to 1.5 billion learners spread over 194 countries (see Fig. 2). To improvise online teaching in place of usual face-to-face schooling was new and represented a big challenge for teachers (Cutri et al., 2020). Change on this scale has not been seen outside wide reform movements in places such as (for example) Australia (Clarke & Ziebel, 2017), the USA (National Council of Teachers in Mathematics [NCTM], 2000), Ontario (Radford & Demers, 2004), and Italy (Unione Matematica Italiana — Commissione Italiana per l’Insegnamento della Matematica [UMI-CIIM], 2001, 2003). Such imposed change may cause teachers to feel a sense of pressure, like a push to conform to new norms (Andrà et al., 2019), and may result in resistance to change with beliefs emerging as a barrier to change (Fives & Gill, 2015).

However, the change provoked by the COVID-19 lockdown was substantially different for many reasons. First of all, the change came without forewarning. Unlike curriculum revisions, teachers had no time to either prepare for it or to argue against it. Secondly, it came as a result of *force majeure* as opposed to a political initiative. Thirdly, there was an absence of the rigid prescriptions about what or how to teach that normally accompany teaching revisions (Clarke & Ziebel, 2017; NCTM, 2000; Radford & Demers, 2004; UMI-CIIM, 2001, 2003). Taken together, during the COVID-19 pandemic, teachers were left somehow free to explore new ways of teaching, with relatively little judgment and no high expectations from stakeholders or institutions.

### Data gathering and method of analysis

Participants in this study were contacted via email and invited to fill in an online questionnaire^1^ composed of both multiple-choice and open-ended questions; both questions and answers were in English, regardless of the mother tongue of the respondents. We sent the email in March 2020 and collected answers until the end of April 2020. The questionnaire had two main sections (S1 and S2). Section S1 contained some questions regarding participants’ teaching experience, inquiring about their confidence with technology and with online teaching, and acted as a window on both their technological knowledge and their beliefs about that. Section S2 had a set of questions focused on teachers’ practice and on the way it changed after governments’ dispositions during the pandemic. In section S2 there were three questions:

- Q1: To what extent are you satisfied with your first week of “distance learning”? (From 1, not at all, to 4, really satisfied). Why?
- Q2: What are the difficulties/potentialities that you faced (during the first week)? Which ones did you expect?
- Q3: Did you change anything with respect to the first week of lessons? If so, what influenced your choice?

In all, 48 university professors from 13 different countries volunteered to answer the online questionnaire about how their teaching had changed during the COVID-19 lockdown. Of these, 18 participants provided detailed answers to all questions, and, as such, they represent the sample of this study. Of these 18 participants, 13 came from Europe, 4 from the Americas, and 1 from Israel. The majority (14 of 18) were teaching a mathematics course, 2 were teaching a physics course, and 2 were teaching a mathematics education course. In this study, we consider only mathematics professors, the majority of whom (*n* = 13) were teaching in classes with less than 100 students at the time of the global lockdown.

The data were analyzed qualitatively. Looking at participants’ accounts of how their teaching changed during the lockdown, we examined if these changes in practice were accompanied by a subsequent change in beliefs and/or knowledge. More specifically, focusing on the answers to Q1, Q2, and Q3, we looked for indicators for each element of the teaching system. For this round of analysis, each of the authors interpreted the answers independently, and then we discussed and compared the analyses until we reached consensus. Table 1 reports examples of indicators after the agreement among the researchers.

**Table 1:** Examples of participants’ answers that contain indicators (underlined) for each element of the teaching system

Practice	Beliefs	Knowledge
- I ask more questions to have more comments from students - I chose to “flip” the classroom - I recorded the videos frequently - Using Document *camera* is similar to writing on the chalkboard	- I feel that the new approach is more fruitful - I find teaching via Zoom to a large group of 21 students is very difficult - Students seem to like my lessons - I think that frontal lessons cannot be replaced for my subject	- I've learned to use better Geogebra - I experimented with writing tablet - With the tablet I am able to use colors, copy and paste formulas

**Fig. 3:**
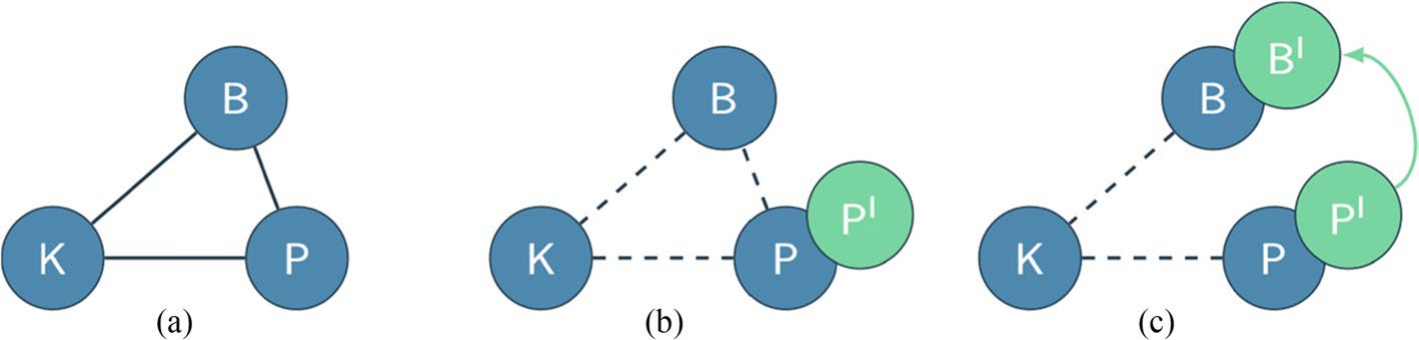
Cindy’s (Cutri et al., 2020) teaching system and its change

Finally, we resorted to the answers to the other questions in both S1 and S2 and employed teaching system analysis. In our analysis, we firstly describe the teaching system before the perturbation of the lockdown, which we represent as the aforementioned connected graph composed of three elements (see Fig. 3a): practice (P), beliefs (B), and knowledge (K). We acknowledge that each element P, B, and K is made of a variety of components; for instance, with K, we refer to the whole body of professional knowledge (Ben-Peretz, 2011); with B, we refer to the belief system as a whole; and with P, we intend all the practices of a teacher. However, for the purposes of the analysis, we distill and report in the analysis only those components that are relevant to the observed changes. A change in P, B, or K is labeled with prime, that is, P^I^, B^I^, K^I^, P^II^, B^II^, K^II^, respectively. To be clear, change happens component-wise, but we intend that the element P, B, and/or K *as a whole* changes. We denote with dashed links the initial teaching system once perturbed (see Fig. 3b). If the change propagates in the system and affects other elements, a directed edge from one element to another denotes the influence of the former on the latter (like in Fig. 3c). Otherwise, the new element stays unconnected.

For example, in Cutri et al.’s (2020) study, Cindy was used to teaching in presence (P) and held a belief (B) that it is a teacher’s duty to fix technical problems in class, but she also had limited technological knowledge (K). We represent this in Fig. 3a. Cindy had to move her teaching online (P^I^, Fig. 3b). She declared that her belief (B) about a teacher’s ability to fix technical problems was challenged when she faced an issue she was unable to solve and a student showed up and fixed it. Hence, Cindy changed her belief about the teacher’s role in the classroom (B^I^). B^I^ can be connected to either old (P) or new practice (P^I^) and to either old or new knowledge. It depends on how the teachers described and lived the change in P, B, or K. In Cindy’s case, it connects to P^I^ (see Fig. 3c) because the change in practice (P^I^) directly caused the perturbation that led to a change in beliefs (B^I^). Eventually, a (new) stable teaching system will form. For example, if Cindy does not like to share power with students, the (old) stable teaching system will be reached, but if she abandons her old belief (B), a (new) teaching system will be established, and the emerged belief becomes hard to change as it connects with practice and knowledge that strengthen the new system composed by P^I^–B^I^–K. In Cutri et al.’s (2020) study, we do not know how the system stabilizes, as data are not reported.

## Results

For all participants in the study, the situation represented in Fig. 3b was the starting point for the change in our analysis, namely, moving from in-person to online teaching represented a significant change in practice. From the answers to Q1, we noticed that all participants were concerned about missing live contact with their students. Indeed, using one professor’s words, “Students don’t participate to the same degree as in the physical classroom.” Moreover, for the huge majority of the participants, the first lecture online went along with a sense of discomfort caused by the lack of non-verbal feedback (“It is hard to get feedback from students, this is my main frustration”), such as eye contact (“Lack of eye contact with students” is the major difficulty for another participant) and head nodding (“real-time students’ feedback is missing”).

The analysis of the mathematics professors led us to identify two scenarios, and in this article, we focus on three participants, fictitiously named as Alejandro, Giovanni, and Carlo. In addition to the features mentioned above, these three participants were chosen for two main reasons. Firstly, because they had different characteristics with respect to their pedagogical and technological knowledge and no experience with online teaching before the COVID-19 pandemic, they experienced the change to their practice most dramatically. Secondly, the three selected cases were so diverse that they allowed both to describe the different scenarios detected and to most clearly exemplify the proposed model. The former reason can be considered as a priori choice, while the latter one is an a posteriori validation of our choice.

The two scenarios that emerged can be briefly described as follows: (i) one in which the teaching system tries to resist change and repair itself (Alejandro and Giovanni) and (ii) one in which the system initiates a change and becomes dynamic (Carlo). These scenarios are not meant as generally exhaustive representations of all possible ways that mathematics professors experienced the lockdown, but they represent different ways a change in practice has produced a move (or not) for the entire teaching system.

### Static scenario: the case of Alejandro

**Fig. 4:**
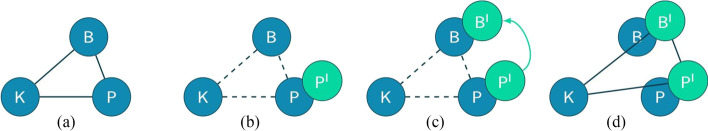
Alejandro’s teaching system and its change

The first case we analyze is that of Alejandro, a mathematics educator who was teaching Calculus I to a medium-sized class (50–100 students) of first year university students in Santiago, Chile. On March 16, 2020, the whole city of Santiago was put under mandatory quarantine due to an increase in cases (Flores Belmar, 2020). When this happened, Alejandro, like all his colleagues, had to migrate his in-person university lectures to digital ones. He described his usual practice as follows: “In my regular classes last year [2019], I formed random groups of 4 students, who solved a problem, when they had doubts they asked me questions and I answered with questions that helped to overcome stagnation. Then the students showed their strategies to the rest of their classmates in a plenary session.” We see that Alejandro’s practice (P) was characterized by participative math lessons. Moreover, Alejandro’s answers in section S1 informed us that he was not very familiar with technological tools, such as online forums, self-produced videos, and online platforms for shared documents. This featured Alejandro’s knowledge (K). Alejandro also reported that he was not confident with the idea of online teaching, because he did not have the right equipment and was worried about the lack of face-to-face interaction with students. Such lack of confidence and worries were part of Alejandro’s belief system (B), as were his explicit beliefs about the importance of student’s participation during lectures. From the complex and rich teaching system of Alejandro, we singled out the elements that emerged in his responses to the questionnaire and could be relevant to understand change, if any. The triplet P–B–K represents the initial configuration of Alejandro’s teaching system, before the pandemic (Fig. 4a).

In the questionnaire (S2), Alejandro answered that, when he was forced to teach online, he prepared the first lesson doing some technical tests, sharing information with colleagues and searching for new tools (for instance, student collaborative system, screencast recording, virtual board). Instead of being problem-based and interactive, Alejandro’s first lesson was transmissive and based on slides. This represented a big shift in Alejandro’s practice (P^I^ in Fig. 4b).

After the first week, Alejandro answered that he was not satisfied with how it went because he did “not know if students were following the explanation.” As a result, he wrote in response to Q2, “[…] group work has not worked very well in online classes.” From these claims, we can infer that two beliefs emerged (B^I^): (i) communication from teacher to students is important (namely, it is relevant for Alejandro that students can follow the explanation); and (ii) group work online is troublesome. These beliefs were induced by the forced change in practice (P^I^). We represent B^I^ and P^I^ as connected to each other in Fig. 4c.

After the first week, Alejandro decided to prepare a new lesson plan and a new schedule of the course, more precisely he declared in Q3: “I changed the pace of my online class via Zoom,^2^ because interaction with students is slower than in person.” What emerged is that he did not change the tools used during lectures and he reported that he did not know how to manage students’ group work; hence, he abandoned it and continued with teacher-led lessons. In other words, we can say that K did not change for Alejandro. In fact, Alejandro was not prompted to look for a way to effectively have group activity online, and he did not fully exploit the potentialities of Zoom, which would have allowed him to create separate rooms for small group activities. We interpret this as lack of (technological) knowledge. Namely, the knowledge did not improve in order to address the necessity to create opportunities for group work online. No K^I^ emerged in Alejandro’s teaching system. Alejandro’s explicit beliefs provoked a tension between importance of group work and impossibility to do that online, with the latter dominating and impacting his practice, making the lecture more teacher-led than desired (P^I^ persisted after the first lecture online). Alejandro’ knowledge did not change even if prompted by the former belief (B) on the importance of students’ participation and problem solving, which was pushed aside by his limited technological knowledge. This forced his practice towards a new static scenario (see Fig. 4d). With B being ignored, and P being impossible during the lockdown, the new teaching system made up of P^I^, B^I^, and K stabilized.

### Static scenario: the case of Giovanni

Giovanni was a researcher in STEM topics (numerical analysis) and had 2 years of experience as a lecturer in Italy. Italy was one of the most affected countries in the pandemic during the spring of 2020 and went through a long national quarantine that lasted more than 60 days in some areas (Anzolin & Amante, 2020). Giovanni worked in Milan, the city that was the epicenter of the pandemic in Italy. Schools and universities closed on February 17, 2020, and remained closed for the rest of the academic year. The second semester was delivered entirely online throughout the whole country. Giovanni taught a large class of about 200 students he had never met in person.

**Fig. 5:**
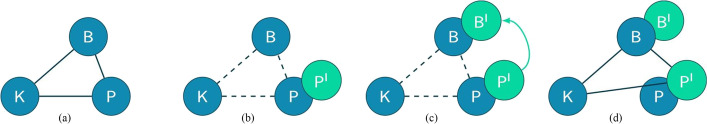
Giovanni’s teaching system and its change

Unlike Alejandro, Giovanni was confident with the idea of online teaching because he had the right equipment and received guidelines from his university. That is, his initial knowledge (K) was formed by these guidelines. Moreover, Giovanni’s answers in section S1 informed us that his practice (P) was featured by traditional transmissive lessons from the front of the room. In section S1, he also claimed that “for teaching is crucial to prepare good notes and the talk.” From this, we infer that he had an explicit belief (B) about the relevance of the material and the importance of the teacher’s lecture. P–B–K in Fig. 5a represents the initial configuration of Giovanni’s teaching system, namely, that frontal lessons were the routine for him, that quality of talk and good notes were the essence of his teaching, and that he had good technological knowledge. Node P^I^ in Fig. 5b resulted from the migration to online teaching.

After the first week of online classes, Giovanni was satisfied because he did not have technical difficulties and “students were active and participated during the lecture.” However, he reported in Q2 that “interaction with students is less than during in-person classes, but they might use the chat to provide me with some feedback. Maybe they would ask more questions than usual.” Giovanni mentioned the chat and students’ participation, which we label B^I^ (Fig. 5c), but we note that, besides wishing for them to use it, Giovanni neither encouraged his students to interact via chat nor did he design the lesson so as to promote students’ interaction. His response to Q3 was, “[I will do the same]. I think that what I did last week, very similar to the in-person lesson, is appropriate.” On one hand, we notice that Giovanni did not improve his pedagogical knowledge, claiming that it was sufficient that the chat was at the disposal of students to use when they needed to interact. On the other hand, and differently from Alejandro, we see that Giovanni was happy with the way online lectures were conducted during the first week. After the first week of lectures, his knowledge did not change, and he was satisfied with the quality of his lecture. His belief B^I^ was ignored, and he kept delivering a transmissive lesson using a virtual whiteboard (P^I^).

Giovanni migrated his practice online (P^I^); a belief about the importance of interaction with students emerged (B^I^) but did not connect to P^I^ because Giovanni was satisfied with his lessons and the belief (B) about the importance and quality of his lecture seemed more relevant for him. We conjecture that B^I^ was also ignored because of Giovanni’s lack of (pedagogical) knowledge. Thus, only practice (P^I^) changed, and the system with P^I^–B–K stabilized as it appears in Fig. 5d.

### Dynamic scenario: the case of Carlo

**Fig. 6:**
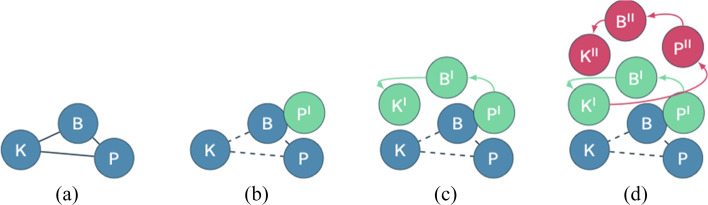
Carlo’s teaching system and its change

Like Giovanni, Carlo worked in Milan and conducted research in STEM topics (numerical analysis). He was an associate professor who had been teaching numerical analysis and calculus for the past 15 years. Unlike Giovanni, however, Carlo was interested in pedagogical aspects. Prior to the COVID-19 lockdown, Carlo taught transmissively using the blackboard and rarely used slides (P). During the pandemic, he had to teach his course entirely online, with a large class of about 200 students he had never met in person. Carlo received guidelines from his institution describing how to conduct lectures: he was told to use a video conference system (such as MS Teams) as a tool to broadcast his lectures. These guidelines formed his initial knowledge (K) in his teaching system. Once Carlo received the guidelines, his main concerns were about the technical difficulties with the use of the videoconference system and about the students’ feedback. Indeed, Carlo was not confident at all with respect to the idea of online teaching, because he liked to have face-to-face interaction with his students and he did “not feel familiar with technological instruments for online live teaching.” From Carlo’s answers, we can infer that he held an explicit belief (B) that it is important to be familiar with technological tools to teach online. As with everyone, COVID-19 brought a change in practice (P^I^). We, thus, represent Carlo’s initial configuration in Fig. 6a and shift in practice in Fig. 6b.

Answering Q1, Carlo wrote that he felt an “unexpected sense of estrangement” due to the fact he “spoke for almost 2 h in front of a computer, without having any kind of feedback.” Even before the first week of online teaching, Carlo realized how important communication was for him, not only the communication from teacher to students, but also from students to teacher. Hence, we identify B^I^ to highlight this change in Carlo’s belief system.

During the first week, he discovered that he “could use the chat [embedded in the online environment] to ask yes/no questions.” After the first week, Carlo searched for a way to interact with his students: “I am resorting to the chat for collecting students’ feedback to the exercises during the lesson, moreover I would also like to discuss orally with students.” Carlo became familiar with the use of the chat, thus improving both his technological and his pedagogical knowledge (K^I^). We notice that, unlike Giovanni, Carlo planned to actively use the chat as part of the lesson and prompted the students to use it. This allowed Carlo to change his practice even more, since he also planned “for the following weeks to be more flexible with [his] schedule and to prepare some small exercises students can do in real-time to be even more active during class” (P^II^). The new change in practice led another belief (B^II^) to develop, namely. the importance of collaboration.

To sum up, at the beginning of the semester, the shift from P to P^I^ for Carlo occasioned a belief (B^I^) to emerge. In order to address it, Carlo acquired new knowledge (K^I^); this allowed him to change his practice (P^II^) that led belief B^II^ to emerge. There was a dynamic situation in which Carlo’s beliefs, practice, and knowledge were changing and influencing each other, as shown in Fig. 6d.

## Discussion

The COVID-19 contingencies, for all the cases reported in this study, provoked a shift in practice from in-person to exclusively online classes (from P to P^I^). This shift presented an opportunity for researchers to observe change initiated by a shift in practice that was neither negotiable nor politically motivated. This is relatively rare in comparison to changes in practice prompted by a change in beliefs or knowledge occasioned by, for example, professional development (Ball, 1988; Feiman-Nemser & Featherstone, 1992). We noticed that this change in practice evoked a change in beliefs, confirming Guskey’s pioneering work (1986).

In the case of Alejandro, the belief about difficulties in online group work (B^I^) overpowered the belief about the value of problem-solving and discussion (B). This, coupled with his relative lack of technological knowledge, had the consequence that Alejandro’s online teaching (P^I^) was very different from his in-person teaching (P): the active participation of students was replaced by transmissive lessons. True, we can question how a professor who had little or no experience with online teaching can learn to manage online group discussion with 50–100 students within a week. However, what is relevant, in our view, is that Alejandro’s way of dealing with the transition to online teaching emphasized the concern on the mathematical content rather than on the way mathematical concepts are shared and used by learners. Cutri et al. (2020) observed that several teachers experience Alejandro’s strain and that “there was an impulse for them to enact a more direct instruction mode when having to rapidly transition their courses online due to the COVID-19 pandemic” (p.11).

Giovanni’s belief about the usefulness of the chat for students’ interaction (B^I^) was not carried forward, because limited pedagogical knowledge did not prompt Giovanni to design lessons that promote the use of the chat by students. We can also say that Giovanni adhered to a transmissive view of mathematics teaching. These two scenarios highlight that (lack of) knowledge may hinder change in teachers’ practice (Ball et al., 2005; Ben-Peretz, 2011). For Alejandro, this lack of knowledge was about online teaching management, and for Giovanni, it was a lack of knowledge about how to promote students’ participation via chat. We can describe both of these as scenarios in which the teaching system resisted the change and repaired the damage (Stigler & Hiebert, 1999). More precisely, the teaching system repaired the damage caused by the lack of knowledge ignoring their beliefs, B in the case of Alejandro and B^I^ in the case of Giovanni. Both Giovanni’s and Alejandro’s cases further confirm the importance of knowledge for teaching, as it emerges in literature, as well as the close and strong relation between beliefs and knowledge, but the power of our model is to describe all this in a systematic way. The cases of Alejandro and Giovanni further show that there is not a single, monolithic static behavior of a teaching system but that there can be different kinds of staticity. In the case of Alejandro, in fact, the new element P^I^ connects with B^I^, whilst the “old” B remains connected to the “old” P; namely, the system has a static behavior in the sense that belief B^I^ emerges to patch the system up and connects P^I^ and the initial K. In the case of Giovanni, the new element B^I^ is ignored; it is as if it is cut out from the teaching system, which connects the new practice with the old B. Attempting a generalization, a teacher can resist change either because a new belief emerges but is connected to a new context for practices that has (in the teacher’s eyes) no connection with her traditional ones or because a new belief emerges but it is discarded by the system. Other kinds of staticity can emerge. For instance, there might be a change in knowledge but without a change in both practice and belief, because the beliefs towards students do not change as reported in Andrà et al. (2020).

In Carlo’s scenario, we observe that the shift in practice (P to P^I^) provoked the emergence of beliefs (B^I^) about the importance of student-to-teacher communication and the acquisition of technological knowledge (K^I^). We described such dynamics as an evolution from P^I^ to B^I^ and then to K^I^. Carlo’s emerging belief (B^I^), coupled with acquisition of technological knowledge (K^I^), drove further change in practice (P^II^). This, in turn, prompted the belief (B^II^), as a dynamical teaching system begins to evolve. Remarkably, Carlo’s appreciation for transmissive teaching was challenged by the emergence of the importance of students’ voices, and, as a result, mathematics teaching became more participative in Carlo’s lessons. And students’ ideas turned out to count more than he believed at the beginning of the online semester. Carlo is a case of teacher change, and change is related to practice, beliefs, and knowledge because teaching has been shown to have a systemic nature. True, in this study, we focused only on P, B, and K as constitutive elements of a teaching system. However, Akkerman et al. (2021) invite us to consider that:

not only educational settings, but also family, peers, and neighborhoods create positions, purposes, and project futures along with ideas about how to engage in school, subjects, how to make educational, vocational, or alternative choices. (p.4)

We thus wonder whether there could be other important elements for the teaching system to describe and explain teacher change or their resistance to change. In either case, on the basis of Akkerman et al.’s (2021) consideration, each teacher’s choice is a choice of success, from their personal point of view. As we elaborate also in the conclusions, Alejandro, Giovanni, and Carlo represent for us three different ways a teacher’s system can respond to change, and they represent three different cases of successful responses to change.

Due to the exploratory nature of our study, a number of issues remain open. First, the small sample of our study does not allow us to describe all the possible scenarios; the three presented cases clearly exemplify our approach, but we aim at conducting a confirmatory study with more participants that will allow us to provide further scenarios. Second, our data consist of the collection of participants reporting on their own teaching experience; therefore, there might be some biases and inaccuracies in data. It would also be necessary to deepen the analysis to better understand the intertwined connection of the three elements of the teaching system and if there are other elements that could be added, as Akkerman et al. (2021) suggested. This can be done with extensive interviews in a follow-up study. Third, a question that remains open at this stage of the research is whether, especially in the case of Carlo, the new P–B–K triplet will either evolve or go back to the initial state when in-person teaching will be restored. For this, we have to wait until the end of the pandemic and possibly go back to Carlo. Paying specific attention to dynamic examples of teaching systems, like Carlo’s, would allow us to better understand important implications of this research for mathematics teachers’ professional development: in particular, to understand the conditions under which change is initiated and maintained, and also to understand why practice is deemed to be difficult to change. The case of Carlo allows us to offer a partial answer, namely, that when a belief emerges in relation to a new practice, if teachers have good knowledge that enables them to adjust their practice so as to follow their beliefs, this creates an opportunity for change. We show this in a concrete example, but it should be further investigated.

## Conclusions

The COVID-19 pandemic created a *storm* in the teaching environment, disrupting old and well-established traditions in teaching and challenging teachers’ routine. In our view, the storm left an open space for change: teachers lost the live contact with their students, and, somehow, this absence created a breach into well-established practices. The ways in which the different teachers in our exploratory study responded to the storm are perfectly captured by this proverb: “When the winds of change blow, some people build walls and others windmills.” Some teachers, like Alejandro and Giovanni, reacted to the storm by building a wall to defend their teaching system, while others, like Carlo, took the storm as an opportunity to build a windmill and power up their teaching system (enriching their knowledge and promoting students’ participation). For those who preferred the windmill, the emergence of new (implicit) beliefs resulted in a continuous evolution of the system. For those who built walls, change in beliefs was not accompanied by change in knowledge, and the COVID-19 lockdown–induced practice became static, apart from the actual migration from in-person to online teaching. We would like to add that there is no judgment on our side on either choice. These teachers represent three ways of living with change, with pros and cons in every case.

Back to the research *problematique*, our data provided evidence that teaching can be thought of as having a systemic nature, and, in line with many previous research findings, the main components of a teaching system are practice, beliefs, and knowledge. Moreover, the P–B–K model has the potential for research to capture many relevant aspects of teaching, such as the importance of teacher’s knowledge in connection to practice and beliefs, as well as to determine whether there are conditions for teacher change or not. By not limiting itself to observation of classroom practices, or to beliefs, or to (lack of) knowledge, the P–B–K model considers teaching as a system made of distinct but intertwined elements, wherein a change in one element comprises a change in the others. As a consequence, we can argue that the model provides insight on how a teacher’s teaching system evolves. On one hand, the teaching system may stabilize due to lack of knowledge and/or resistance of the belief system; on the other hand, the teaching system may spin until a new, different teaching system stabilizes.

## Footnotes

1 The questionnaire, in English, is available at this link https://forms.gle/isrXEqU4oQis5tbf7.

2 Zoom is one of the video call systems. We underline that Zoom removed the restriction of 40 min for the free account during the COVID-19 pandemic for educational purposes.
